# Tumor-Associated Macrophages (TAMs) in Colorectal Cancer (CRC): From Mechanism to Therapy and Prognosis

**DOI:** 10.3390/ijms22168470

**Published:** 2021-08-06

**Authors:** Hui Wang, Tian Tian, Jinhua Zhang

**Affiliations:** 1 National Center for International Research of Bio-Targeting Theranostics, Guangxi Key Laboratory of Bio-Targeting Theranostics, Collaborative Innovation Center for Targeting Tumor Diagnosis and Therapy, Guangxi Talent Highland of Bio-Targeting Theranostics, Guangxi Medical University, Nanning 530021, China; wanghuigxykdx@163.com; 2 College of Life Science and Bioengineering, Beijing Jiaotong University, Beijing 100044, China

**Keywords:** colorectal cancer, tumor microenvironment (TME), macrophage, polarization, therapy, prognosis

## Abstract

Colorectal cancer (CRC) is a malignant tumor in the digestive system whose incidence and mortality is high-ranking among tumors worldwide. The initiation and progression of CRC is a complex process involving genetic alterations in cancer cells and multiple factors from the surrounding tumor cell microenvironment. As accumulating evidence has shown, tumor-associated macrophages (TAMs)—as abundant and active infiltrated inflammatory cells in the tumor microenvironment (TME)—play a crucial role in CRC. This review focuses on the different mechanisms of TAM in CRC, including switching of phenotypical subtypes; promoting tumor proliferation, invasion, and migration; facilitating angiogenesis; mediating immunosuppression; regulating metabolism; and interacting with the microbiota. Although controversy remains in clinical evidence regarding the role of TAMs in CRC, clarifying their significance in therapy and the prognosis of CRC may shed new light on the optimization of TAM-centered approaches in clinical care.

## 1. Introduction

Colorectal cancer, whose abnormal cells grow in the colon or the rectum of the large intestine, is the third most common malignant tumor worldwide. The global incidence of colorectal cancer (CRC) has increased in recent years. According to the global cancer statistics for 2020 released by the International Agency for Research on Cancer (IARC), there were approximately 1.9 million new cases of CRC and 940,000 cancer deaths worldwide in 2020; its global mortality rate ranks second [1]. Some Asian countries, such as Japan and Malaysia, also have high CRC incidence. In China, the incidence of CRC has risen to become the fourth most common malignancy with the fifth highest mortality rate [2], making CRC a major public health issue.

The main risk factors of CRC are increasing age and genetic, lifestyle, and environmental factors. Genetic and epigenetic alterations inside the cell—such as the activation of oncogenes and proliferative signals from the abnormal microenvironment surrounding the cell—play the intrinsic role, while lifestyle or environmental factors such as obesity, inadequate exercise, tobacco or alcohol use, and processed meat consumption constitute the exogenous causes of CRC, interacting synergistically with the endogenous factors to promote CRC occurrence and development [3,4].

Over the past two decades, increasing evidence has shown that the tumor microenvironment (TME) plays an equally significant role in tumor initiation, progression, and metastasis as the genetic and epigenetic changes in cancer cells. The components of the tumor microenvironment include all of the nonmalignant stroma cells inside the tumor other than the tumor cells, including fibroblasts, endothelial cells, immune cells, and platelets [5,6]. Paget proposed the “seed and soil” theory in 1889 and conducted an in-depth analysis of the molecular characteristics of “seeds” (cancer cells) [7,8,9,10,11]. By studying the “soil” formed by cancer cells and host immune cells, scientists found that immune cells usually bind to cancer cells and obtain specific biological phenotypes via interactions with them. As a result, the TME is a unique environment which develops alongside tumor progression. It is now widely recognized that neglection of the complex changes in tumor microenvironment during tumor development is one important reason for the failure of current targeted therapies against tumor cells. Therefore, new therapeutic strategies targeting the component cells of the TME can be combined with traditional treatments to benefit CRC patients in individual medicine.

Tumor-associated macrophages (TAMs) are a major component of the immune cells of the TME. They play a prominent role by secreting cytokines and chemokines and coordinating with inflammatory mechanisms to promote tumor development, invasion, metastasis, immunosuppression, angiogenesis, and drug tolerance [12,13,14,15,16,17,18,19]. Different subtypes of TAMs have different functions, which can be dynamically changed in response to various signals from cancer cells or the TME. Studies have shown that TAMs are associated with poor prognosis in most solid tumors; however, their role is slightly more complicated in CRC, in which reeducating the polarization of TAMs may facilitate tumor immunotherapy [17,20,21,22,23,24]. The current review focuses on the phenotypic polarization of TAMs in CRC, the underlying functional mechanisms, and how these mechanisms can be used as potential targets for the treatment and prognosis of CRC.

## 2. TAM Origin and Phenotypic Polarization

Macrophages, a component of the mononuclear phagocytic system (MPS), play a crucial role in maintaining the innate immune response, tissue homeostasis, and inflammation [25]. Bone marrow-derived macrophages (BMDMs) originate from hematopoietic stem cells which generate myeloid progenitors, while tissue-resident macrophages (TRMs) develop from precursor cells of macrophages in different tissues during embryonic development. The progenitor cells receive inflammatory mediator stimulation signals and differentiate respectively into mature macrophages with different phenotypes [26,27]. For example, after birth the embryonic macrophages in the colon are substituted quickly by monocytes from hematopoietic stem cells (HSCs). Studies have shown that TAMs predominantly come from circulating inflammatory monocytes, which can be recruited by multiple chemokines including CC-chemokine ligands 2 and 5 (CCL2 and CCL5) and cytokines colony stimulating factor 1(CSF1) [28,29]. Tissue-specific embryonic-derived resident macrophages that infiltrate tumor tissues are also an important source of TAMs; however, the situation may vary according to different tissues or organ specificities [30,31,32,33]. Various chemokines induce monocyte-related myeloid-derived suppressor cells (M-MDSCs) into TAMs [34]. Since most of this origin-tracing research on macrophages was performed in animal models, the actual situation in human cancer remains to be elucidated [17].

Through specific differentiation, macrophages can evolve into two different polarization states: classically activated M1 (proinflammatory) and, alternatively, activated M2 macrophages (anti-inflammatory) [12,35]. M1 macrophages have the capability to promote the Th1 response and engulf and kill target tumor cells [36]. Studies have shown that M1 macrophages are induced by cytokines such as interferon γ (IFN-γ), lipopolysaccharide (LPS), or tumor necrosis factor-α (TNF-α) [37]. In the TME, M1 TAMs secrete interleukin-6 (IL-6), interleukin-23 (IL-23), reactive oxygen species (ROS), and other inflammatory mediators that participate in the inflammatory response and exert antitumor immunity. In contrast, under the induction of interleukin-4 (IL-4), interleukin-10 (IL-10), interleukin-13 (IL-13), or glucocorticoid-induced differentiation, M2 macrophages secret anti-inflammatory cytokines such as IL-10 and interleukin-1β (IL-1β), and promote angiogenesis, tissue remodeling, injury repair, and tumor initiation and progression [18]. M2 macrophages are typically divided into four subtypes according to their different stimuli: M2a (typically induced by IL-4 and IL-23); M2b (induced by immune complexes with IL-1β or LPS); M2c (induced by IL-10, transforming growth factor beta, and glucocorticoids); and M2d (induced by IL-6) [12,38]. TAMs primarily have the phenotype of M2-like macrophages in the TME and promote tumor immunosuppression by promoting tumor angiogenesis or by indirectly inducing interactions between immune cells in the TME [39]. It is interesting that the M1 and M2 polarization states can switch from one to the other, implying that macrophages may be a potential therapeutic target [40,41]. In fact, the polarization state of macrophages, especially in disease, is complicated; macrophages with both M1 and M2 characteristics, and those with neither of the two, also exist in the TME.

## 3. The Functional Mechanism of TAMs in CRC

**Figure 1:**
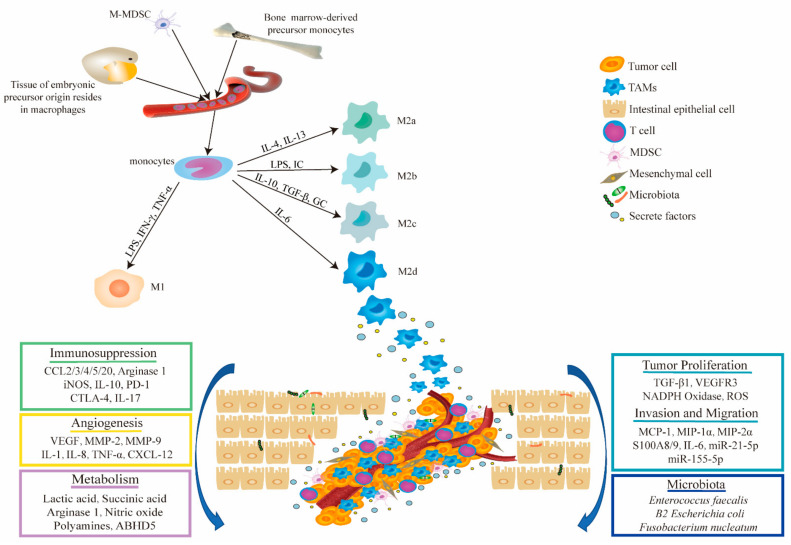
Origin and phenotypic polarization of TAMs and their functional mechanism in CRC. TAMs mainly originate from monocytes which arise from bone marrow-derived precursors, tissues of embryonic precursors, and myeloid-derived suppressor cells (M-MDSC). TAMs can be polarized into classically activated M1 (pro-inflammatory) and alternatively activated M2 (anti-inflammatory) macrophages. TAMs promote tumor occurrence and malignant progression by stimulating tumor-related angiogenesis; promoting tumor cell proliferation, invasion, and metastasis; inhibiting the antitumor immune response; regulating metabolism; and interacting with microbiota in the GI tract. LPS, lipopolysaccharide; IC, immune complex; GC, glucocorticoid. The legend for different cell types is shown in the upper right.

The gastrointestinal tract holds the largest number of macrophages, which play a significant role in clearing pathogens, regulating inflammatory responses, maintaining homeostasis, and adjusting insulin sensitivity [42,43]. Upon stimulation by the external environment, macrophages recruit monocytes from the blood to the tumor site and polarize them into TAMs, which are the most abundant immune cells in the TME of CRC. TAMs can interact with tumor cells by means of exosomes or the secretion of several cytokines to promote the proliferation, invasion, migration, and angiogenesis of the tumor cell. TAMs recruit regulatory T cells (Tregs) by secreting the chemokine CCL2, suppress the antitumor immune response of T cells, and interrupt immune cell interactions, resulting in the immunosuppressive microenvironment of CRC [25,44,45]. In addition, TAMs also function through different metabolic pathways and interactions with the microbiota in CRC. We further explain these different functional mechanisms in detail in the following section. The origin, phenotypic polarization, and multifaceted role of TAMs in the TME of CRC are shown in Figure 1.

### 3.1. Regulation of Phenotypic Polarization of TAMs in CRC

Since tumors initiate in a chronic inflammatory environment from the stroma of the epithelia, TAMs are mainly proinflammatory M1 macrophages in the early stage, which produce reactive oxygen and nitrogen species, leading to oncogene activation in the nearby epithelia [44]. After neoplasia occurs, more bone marrow-derived monocytes from the blood vessels are recruited to the site of the tumor, where they secrete growth factors and chemokines such as CCL2, CCL5, vascular endothelial growth factor (VEGF), and transforming growth factor beta (TGF-β) [44,46,47]. Zhu et al. found that *fibrinogen 2* (*Fgl2*) is essential for immune regulation of the colon in an inflammatory state. Loss of *Fgl2* induces the polarization of M1 macrophages, thereby inhibiting the formation of colitis-associated cancer (CAC) [20]. Gao, L. et al. reported that the expression of *phospholipase D4* (*PLD4*) in TAMs promotes the activation of M1 macrophages, resulting in an antitumor effect on colon cancer cells, and that its expression is related to the clinical stage of colon cancer [48]. Cheng, Y. et al. confirmed for the first time that PKCα acts as a tumor suppressor in the intestine, acting through the MKK3/6-P38 signaling pathway to promote IL12/GM-CSF-mediated M1 polarization and inhibit the growth of mouse colon cancer [49].

Through multiple signaling pathways, tumor cells make use of TAMs to support advanced CRC growth and progression. TAMs are inclined to convert into anti-inflammatory and cancer-promoting M2 phenotypes with tumorigenic activity. In vitro coculture of a macrophage cell line (THP-1) and a colon cancer cell line (HCT8 or HCT116) significantly increased the number of M2 TAMs. Further mechanistic studies showed that colon cancer cells secrete EGF to promote the M2 polarization of TAMs through the EGFR/PI3K/AKT/mTOR pathway [24]. Similarly, when the PI3K/AKT pathway is activated, paxillin can promote the proliferation and invasion ability of colon cancer cells by inducing the polarization of M2 macrophages, thereby accelerating tumor progression [50]. Obesity-induced IL-6 expression promotes the polarization of M2 macrophages and induces secretion of the chemokine CC-chemokine ligand 20 (CCL20) in the CAC microenvironment; CCL20 recruits CC-chemokine receptor 6 (CCR6)-expressing B cells and γδ T cells via chemotaxis, leading to CAC progression [51]. It has been reported that exosomal miR-155-5p derived from M2 macrophages can accelerate the occurrence and development of colon cancer via its effect on ZC3H12B-mediated IL-6 stability [52]. Exosomal miR-1246, secreted by colon tumor spheres, increased the proportion of M2 polarized macrophages in vitro [53]. In addition, SLC7A2 deletion in colonic epithelial cells significantly increased the levels of the proinflammatory cytokines/chemokines IL-1β, C-X-C motif ligands 1, 2, and 5 (CXCL1, CXCL2, and CXCL5), interleukin-3 (IL-3), and CC-chemokine ligands 3 and 4 (CCL3 and CCL4) in tumors and stimulated the polarization of tumorigenic M2 macrophages [54]. Furthermore, it was reported that both interferon regulatory factor (IRF)/STAT signaling and nuclear factor-κB (NF-κB) play crucial roles in regulating macrophage plasticity [55,56,57]. TAMs induce IL-10 to promote tumor growth, and secrete VEGF to promote tumor angiogenesis, via the STAT3 pathway [58,59,60].

### 3.2. TAMs Promote Tumor Proliferation, Invasion, and Migration

In the TME, TAMs and tumor cells promote tumor cell proliferation through the secretion of cytokines. In vitro studies confirmed that colon cancer cells upregulated the expression of *RGC-32* in macrophages by secreting TGF-β1, and RGC-32 promoted the migration of macrophages and further accelerated the proliferation of colon cancer cells [61]. Yu, X. et al. reported that overexpression of C-X-C-motif receptor 4 (CXCR4) in the intestinal epithelial mucosa can promote epithelial–mesenchymal transition (EMT) and macrophage infiltration in colonic tissue, leading to colitis-associated tumorigenesis and progression [62]. Tacconi, C. et al. found that TAMs expressed VEGFR3, inhibited antitumor immunity, and promoted primary colorectal cancer growth through the VEGFC/VEGFR3 axis [63]. Furthermore, by coculturing TAMs and CT26 colon cancer cells in vitro, it was found that the oxidative stress regulated by TAMs can affect the proliferation of colon cancer cells. TAMs can maintain the level of reactive oxygen species (ROS) by regulating the activity of NADPH oxidase, thereby maintaining the redox state of the TME and promoting tumor cell proliferation [64].

Tumor invasion and metastasis are the main reasons for over 90% of cancer patient deaths. Many studies have shown that the tumor-promoting effect of TAMs can induce the growth and metastasis of colon cancer cells [65,66]. Phinney, B.B. et al. proved that TAMs secrete the chemokines monocyte chemotactic protein-1 (MCP1) and macrophage inflammatory proteins 1α and 2α (MIP-1α and MIP-2α) via the MAPK-activated protein kinase 2 (MK2) pathway, which induces tumor cell growth and invasion in vitro [67]. Lim, S.Y. et al. found that TAMs induce the expression of S100A8/A9 messenger RNA (mRNA) in the colon cancer TME in an ERK-dependent manner and can stimulate tumor migration [68]. Wei, C. et al. found that TAMs secrete IL-6 to induce EMT, which enhances CRC migration and invasion by regulating the JAK2/STAT3/miR-506-3p/FoxQ1 axis [69]. STAT3 promotes the nuclear localization of β-catenin which further enhances growth regulation [59]. In addition, HCT116 and HT29 colorectal cancer cells increase the level of vimentin expressed by M2 macrophages but decrease the level of E-cadherin and demonstrate enhanced invasion ability [66]. M2 macrophage-derived exosomes miR-21-5p and miR-155-5p were transferred to colorectal cancer cells and downregulated BRG1 expression by binding to its coding sequence, accelerating colorectal cancer metastasis [70]. *Six1* overexpression in MC38 recruits TAMs by increasing the expression of macrophage-specific colony stimulating factors CCL2 and CCL5, further promoting the growth and metastasis of CRC and remodeling the tumor matrix [71].

### 3.3. TAMs Enhance Angiogenesis in CRC

The blood supply of tumor cells plays an important role in the occurrence and metastasis of tumors, and tumor angiogenesis is a key step in tumor progression. TAMs are also major players in the regulation of tumor angiogenesis in CRC [72,73,74,75]. According to the study of Badawi et al., there was a significant correlation between macrophage infiltration and microvessel density in malignant CRC [76]. TAMs release a wide range of cytokines, such as VEGF, IL-1, interleukin-8 (IL-8), TNF-α, and matrix metalloproteinase (MMP), which synergistically regulate endothelial cells, matrix remodeling, and vascularization in a spatio-temporal manner in CRC angiogenesis [77,78].

VEGF secreted by TAMs plays an important role in promoting tumor angiogenesis in CRC [79]. TAMs in colon cancer can secrete IL-1, IL-6, and TNF-α, and activate the nuclear factor NF-κB signaling pathway in the vascular endothelium to produce VEGF, which in turn promotes angiogenesis and alters the TME [80]. Macrophages can facilitate endothelial cell migration by secreting thymidine phosphorylase and various MMPs to promote angiogenesis [28,81,82]. It has been reported that GPR35, expressed on macrophages, acts as a major receptor for tumor growth by promoting tumor angiogenesis and MMP activity, mainly due to on Na/K-ATPase-dependent activation [83]. IL-10 stimulates macrophages to secrete the soluble factors MMP-2 and MMP-9, which effectively promote cancer cell-induced angiogenesis in vivo [84]. By adoptive transfer of WT macrophages into MK2 KO mice, Suarez-Lopez, L. et al. demonstrated that MK2 signaling/angiogenesis is inherent in macrophages; MK2 regulates CXCL12 expression in TAMs, promoting angiogenesis and progression of cancer cells [85]. Luput, L. and his team demonstrated that TAMs can enhance the expression of angiogenic proteins in the TME by regulating the activity of NADPH oxidase, which maintains the redox status and angiogenic capacity in the TME of CRC [64].

### 3.4. TAMs Regulate Immunity in TME of CRC

The TME is mainly composed of stromal cells and immune cells such as macrophages, T lymphocytes, natural killer (NK) cells, dendritic cells (DC), neutrophils, and myeloid-derived suppressor cells (MDSCs) [5,86,87]. As the main component of the TME, the initial mechanism of TAMs is to recruit and activate T cells and NK cells by presenting tumor antigens, producing chemokines and cytokines, inhibiting the immune microenvironment of colon cancer, and exerting an immunosuppressive effect [88]. The release of chemokines mediated by TAMs, such as CCL2, CCL3, CCL4, CCL5, and CCL20, further contributes to Treg cell recruitment into the TME, and TAMs suppress the antitumor effects of T cells and NK cells [12,89,90].

TAMs suppress T cells in direct and indirect ways. TAMs inhibit T cell proliferation through arginine metabolism regulated by arginase1, iNOS, and oxygen or nitrogen radicals [29,91,92]. TAMs directly inhibit CD8^+^ T cell cytotoxicity through myeloid-specific NOD1 signaling via the release of arginase 1, which promotes immunosuppression and the tumor-permissible tissue microenvironment in CRC development [17,93]. TAMs produce IL-10, which can suppress CD8^+^ T cell activation by decreasing CD8 protein and T cell receptor colocalization [94]. Additionally, CD206, which is highly expressed in TAMs, can inhibit CD45 phosphatase activity, resulting in impairment of cytotoxicity in CD8^+^ T cells [95]. Studies demonstrated that TAMs may regulate T cell recruitment and restrict T cell localization to indirectly inhibit their activity [17].

TAMs highly express the ligands PD-1 and CTLA-4 (along with PDL1, B7-H1, and other ligands); suppress the cytotoxic function of T cells, NKT cells, and NK cells; and further reduce the body’s ability to kill colon cancer cells [96]. Peritoneal macrophages secrete IL-17, which enhances G-MDSCs accumulation, increases the proportion of Th17 cells, and ultimately promotes CAC development [97]. Furthermore, mutant p53 regulates macrophages through exosomes miR-1246 to increase the activity of TGF-β and promote the anti-inflammatory immunosuppression of macrophages [98].

### 3.5. Metabolic Alterations of TAMs in CRC

Metabolism regulates the differentiation, mobilization, phenotypic polarization, and function of macrophages. The metabolic pathways are significantly different among heterogeneous macrophages. In cancer, the macrophage metabolism reprogramming induced by cytokines and other mediators from tumor cells and the TME involves changes in metabolism-related enzymes, metabolites, and metabolic pathways [99,100,101].

Aerobic glycolysis, also known as the “Warburg effect”, is typical in metabolism of growing tumor cells. During aerobic glycolysis, glucose is metabolized into lactate that is secreted by cancer cells, inducing VEGF and arginase 1 (ARG1) expression in TAMs. This process results in macrophage recruitment and phenotypic polarization towards the M2 macrophage state, mediated by hypoxia inducible factor-1α (HIF1α) [102]. Aerobic glycolysis is also typical in proinflammatory M1 macrophages, while slower aerobic glycolysis—mainly fatty acid oxidation—is characteristic in anti-inflammatory M2 macrophages [100,103]. Classically activated M1 macrophages induce glycolysis via the AKT/mTOR/HIF pathway and use the low-efficiency aerobic glycolysis pathway to enact the host defense against pathogens, including producing MAPK-mediated ROS to eliminate bacteria or tumor cells [104]. This metabolic pathway is similar to the mechanism in tumor cells and requires the upregulation of genes controlled by hypoxia inducible factor 1α (HIF-1α) to activate the transcription of glycolytic genes [105]. Metabolic changes enable M1 macrophages to produce lactic acid, succinic acid, and nitric oxide (NO), which are essential to their function [106,107]. Alternately, activated M2 macrophages participate in the uptake of fatty acids and the reprogramming of lipid metabolism by extracting, breaking down, and/or storing free fatty acids released by fat cells as triglycerides [108]. Bronte, V. and colleagues unveiled a novel mechanism by which altered glycosylation in epithelial cells promotes the pathogenesis of ulcerative colitis and colitis-associated colon cancer via the production of IL13 and CC-chemokine ligand 17 (CCL17) by M2-polarized macrophages [109]. Additionally, downregulation of the peroxisome proliferator-activated receptor-γ (PPAR-γ) pathway in TAMs leads to increased secretion of itaconate, which functions as a regulator of M2 macrophages and promotes tumor progression [110,111]. Recently, our group has demonstrated that myD88 in myofibroblasts facilitates the secretion of osteopontin (OPN) and promotes the M2 polarization of macrophages resulting in STAT3/PPARγ signaling pathway activation and CRC development in a murine model [112].

The key difference between the M1 and M2 phenotypes is the way arginine is metabolized [113]. M1 macrophages achieve arginine metabolism by upregulating nitric oxide synthase (iNOS) expression to produce citrulline and nitric oxide [114]. M2 macrophages produce urea, polyamines, and ornithine through ARG1, which are essential for the function of macrophages in wound healing [115]. Ornithine secreted by M2 macrophages promotes tumor growth and metastasis by activating tumor cell IL-33 expression [116]. Glucose metabolism promotes tumor progression by inhibiting cell death and exerts anti-inflammatory effects in ischemic gut mucosa. Huang, C. Y. et al. found that exposure to cancer-derived glucose metabolites downregulates cell death-inducing TNF-α and upregulates the Th1/17-polarizing IL-12/IL-23 axis in macrophages, ultimately contributing to tumor progression [117]. Furthermore, as a coactivator of adipose triglyceride lipase (ATGL), ABHD5 participates in ATGL catabolism by catalyzing the hydrolysis of intracellular triglycerides [118]. Studies have revealed that the triglyceride metabolism of TAMs is involved in tumor processes. ABHD5 expressed by TAMs promotes the growth of colorectal cancer by inhibiting the production of spermidine by SRM. This suggests that ABHD5 in TAMs may be a target for CRC treatment [119].

### 3.6. Crosstalk between Macrophages and the Microbiota in CRC

Substantial evidence has shown that intestinal flora promotes the development and progression of CRC through direct action and bystander immunomodulatory activity [120,121,122]. Bacterial dysbiosis weakens the barrier of the intestinal tract, which is conducive to bacterial translocation and macrophage activation, thereby establishing chronic tumorigenic inflammation [123]. Studies have shown that there is a close relationship between macrophages, gut bacteria, and tumor promotion [121,124]. LPS in the gut microbiota triggers the regulation of monocyte-like macrophages (MLM) accumulation depending on chemokines, and generates an inflammatory milieu to promote colitis-associated tumorigenesis [125]. Macrophage depletion completely eliminates the tumor-promoting effect of intestinal bacterial dysbiosis, which proves that bacteria needs macrophages to promote tumor development [126]. Additionally, the microbiota changes related to CRC progression are also driven by macrophages.

In CRC patients, total bacterial diversity and abundance in the gut are reduced, resulting in an enrichment of selected bacterial species that enhance macrophage-driven tumorigenic activity. For instance, *Fusobacterium nucleatum* facilitates M2 macrophage and MDSC recruitment, forming an immunosuppressive TME for tumor development and progression. *Fusobacterium nucleatum* maintains M2 polarization through involvement of TLR4 following activation of IL-6/STAT3/c-MYC signaling. Furthermore, *Fusobacterium nucleatum* infects macrophages leading to IDO upregulation on the cell surface, which indicates that *Fusobacterium nucleatum* may trigger macrophages to drive immunodepression [127,128,129]. *Enterococcus faecalis* and B2 *Escherichia coli* exert tumorigenic activity through the bystander effects induced by macrophages. These bacteria colonize colon tumors and stimulate macrophages to produce protumoral factors such as cycoxidase-2 (COX-2) to support key processes in tumor progression [130,131]. Previous studies have shown that *Enterococcus faecalis* can polarize colonic macrophages into the M1 type, which have been shown to cause aneuploidy and chromosomal instability in colon cancer epithelial cells [132,133]. Similarly, when exposed to *Enterococcus faecalis* infected macrophages, mouse primary colonic epithelial cells strongly expressed stem cell markers. The interaction between *Enterococcus faecalis* and macrophages directly contributes to the precancerous transformation of primary colonic epithelial cells [133]. *Fusobacterium nucleatum* is selectively recruited by tumor-associated neutrophils, myeloid-derived suppressor cells, and M2 macrophages during the development of colon cancer, promoting tumor growth [128]. Although most studies have focused on tumor-causing bacterial species, some bacterial species can slow down the progression of tumors through promotion of the immune response. *Helicobacter pylori* infection reduced the infiltration of M2 TAMs into CAC tumors and downregulated the expression of pro-inflammatory and protumorigenic factors such as TNF-α, IL-1β, IL-6, and IL-23 in the tumors of CAC mice [134].

In addition to bacteria, microbiota also include the virome and mycobiome, whose alterations are proven to contribute to CRC. Understanding of the mechanism of this action is still in the initial stage, and more evidence needs to be generated [135].

## 4. Potential Applications of TAMs in Therapy of CRC

Given the significance of TAMs in CRC—as discussed in the previous section: TAMs facilitate tumor proliferation, invasion, migration, and angiogenesis; suppress antitumor immunity; regulate metabolism; and interact with the microbiota—there has been growing interest in new strategies that target TAMs in CRC treatments. Although preclinical studies have obtained some promising evidence that supports a combination of these approaches with traditional methods such as chemotherapy or radiation therapy, new therapeutic approaches targeting TAMs should be carefully evaluated for efficacy and safety in clinical trials.

### 4.1. Blocking Monocyte Infiltration in CRC

Blocking the infiltration of mononuclear cells in tumor-related inflammatory tissues is a promising strategy for the treatment of primary tumors. Chanmee, T. et al. confirmed that colon cancer TAMs induce an enhanced expression of the transcription factors HIF-1, CXCL-12, and CXCR4 in the hypoxic TME environment. Targeting the HIF-1/CXCR4 pathway blocks the accumulation of TAMs [12]. Furthermore, NT157 represents a new class of anticancer drugs that targets both the IGF-1 receptor (IGF-1R) and the STAT3 oncogenic signaling pathway, exerting an inhibitory effect on tumor cells. Studies have shown that NT157 inhibits the expression of tumorigenic cytokines, chemokines, and growth factors, such as IL-6, IL-11 and IL-23, CCL2, CCL5, CXCL7, CXCL5, intercellular adhesion molecule-1 (ICAM1), and TGF-β, thereby inhibiting TAMs in the TME [136]. Mantovani et al. found that TAMs derived from monocytes in colon cancer have the ability to differentiate. Thus, a combination therapy that blocks differentiation is urgently needed to effectively target these cells. TNF-γ can induce monocyte or macrophage recruitment to the TME of colon cancer and inhibit their differentiation into TAMs in vivo [137].

### 4.2. Repolarizing TAMs

The plasticity of macrophages allows researchers to re-educate TAMs. Since TAMs mainly exhibit the M2 phenotype and promote angiogenesis and immunosuppression [138,139], TAMs can be re-educated by inducing polarization from the M2 to the M1 phenotype. For instance, Georgoudaki, A.M. et al. investigated the effect of immune checkpoint therapy by inhibiting the expression of macrophage receptor with collagenous structure (MARCO) by TAMs, which repolarized TAMs to the M1 type in a mouse MC38 colon cancer model and induced antitumor activity [140]. As a small-molecule immunotherapy, tasquinimod reduces the immunosuppressive potential of the TME by altering the number and frequency of tumor-infiltrating myeloid cells [141]. Olsson, A. et al. found that tasquinimod targets early-stage tumor-infiltrating myeloid cells and induces phenotype switching from the proangiogenic and immunosuppressive M2-like phenotype to the proinflammatory M1-like phenotype, which alters the TME to promote immunomodulation, prevent angiogenesis, and inhibit metastasis [142].

As evolutionarily conserved tumor suppressors, T2 RNases can inhibit tumor growth in vivo by balancing the M1/M2 macrophage ratio in tumors and recruiting adaptive antitumor CD8^+^ T cells [143]. Furthermore, Halama, N. and his colleagues also confirmed that inhibiting CCR5 can repolarize the phenotype of TAMs from M2 to M1 by regulating the STAT3/SOCS3 signaling pathway in TAMs, thereby exerting antitumor effects in a phase I clinical trial of patients with CRC liver metastases [144].

### 4.3. Targeting TAMs in Immunotherapy

Immunotherapy has gradually become an effective method of antitumor therapy. Immunotherapy for CRC mainly includes immune checkpoint inhibitors, T cell therapy, and autologous tumor vaccines [145,146,147,148]. Among these approaches, immunotherapy targeting immune checkpoint inhibitors has been clinically verified with corresponding targets, such as CTLA-4, PD-1, and PD-L1 [17,37,149]. CTLA-4 is a coinhibitory molecule expressed by T cells that induces an inhibitory signal via binding to the ligand CD80/86 on adenomatous polyposis coli (APC) [150]. PD-1 on T cells is an immunosuppressive receptor, and plays an important role in inhibiting antigen-specific T cell responses when binding to its receptor PD-L1 [151]. Korehisa, S. et al. found that in patients with colon cancer with high microsatellite instability, PD-L1 is mainly expressed on aggressive front-end tumor cells and by CD68/CD163-positive M2 macrophages, and PD-L1 expression is accompanied by characteristics such as poor tumor differentiation, lymphatic invasion, and tumor budding [152]. Gordon, S. R. and his team found that the expression of PD-1 by TAMs increases as the disease progresses. Further experiments showed that PD-1 expression was negatively correlated with the phagocytic ability of TAMs, and that blocking PD-1-PD-L1 in vivo increased the phagocytic ability of macrophages, reduced tumor progression, and prolonged survival in mice [153].

## 5. TAMs and Prognosis in CRC

The role of TAMs seems to be complicated in regard to colon cancer progression, as they are reported to perform both tumor-suppressive and tumor-promoting activities [154]. Some studies have shown that TAMs are associated with better CRC patient prognosis, while others have associated TAMs with poor prognosis. A summary of the related literature is shown in Table 1.

Some studies have shown that CD68^+^ TAMs are mostly distributed in CRC tumor stroma, mainly along the front edge of the invasion, and CD68^+^ TAMs infiltrated into this site can improve the prognosis of CRC patients [155,156,164,165]. Feng, Q. et al. recruited two independent cohorts of consecutively enrolled patients at one medical center with pathological stage II colon cancer after radical resection. In both cohorts, adjuvant chemotherapy significantly prolonged the recurrence-free survival (RFS) and overall survival (OS) rate of patients with a high CD206/CD68 ratio. This suggests that the CD206/CD68 ratio is probably a better biomarker for prognosis and prediction of stage II colon cancer after adjuvant chemotherapy [157]. However, TAM infiltration alone was not highly significant in prognostic analysis, while the presence of both CD68- and VEGF-expressing TAMs was predictive of better survival rates in stage II and stage III colon cancer patients [158]. In addition, Najbauer, J. et al. found that high M1 macrophage infiltration is correlated with a better prognostic situation in CRC patients in a stage-dependent manner [159].

Nevertheless, different types and locations of TAMs have different prognostic significance for CRC patients. Infiltration of CD68^+^ TAMs and M2 TAMs is associated with poor CRC prognosis [69,70,166]. Infiltration of CD68^+^/iNOS^−^ TAMs in the tumor stroma is a negative prognostic factor [160]. An increased CD163^+^/CD68^+^ ratio in the tumor invasive front (TF) was positively correlated with shorter RFS and OS rates in CRC [66]. In addition, an increase in the proportion of M2/M1 type TAMs was positively correlated with liver metastases in patients with colorectal cancer [161]. In a retrospective study of 123 patients with advanced CRC who were treated with bevacizumab combined with chemotherapy, the RFS and OS rates of CRC patients with low tumor interstitial CD68^+^ TAMs were significantly higher. This suggests that an increase in the number of CD68^+^ TAMs infiltrating the tumor stroma may reduce the efficacy of bevacizumab combined with chemotherapy in patients with advanced CRC [162]. In addition, Herrera, M. et al. demonstrated that the combination of FSP-1^+^ CAFs (cancer-associated fibroblasts) and CD163^+^ M2 TAMs was associated with poor survival rates more significantly than when these markers were studied alone [163].

## 6. Discussion and Future Perspectives

As a progressive cancer, the incidence of CRC is increasing worldwide and its pathogenesis involves multiple complicated factors. TAMs are vital components in the TME of CRC, and multifarious signaling pathways and cells in the TME affect the differentiation of TAMs. In the current review, we discussed the phenotypical polarization, functional mechanism, and potential application of TAMs in CRC therapy and prognosis. Generally, TAMs perform their significant roles by accelerating tumor proliferation, invasion, and migration; facilitating angiogenesis; suppressing antitumor immunity; transforming metabolic profiles; and interacting with microbiota in the colon. Based on this analysis of the working mechanism of TAMs in CRC, therapies targeting TAMs can be accordingly clarified into: those limiting monocyte infiltration, those reprogramming polarization of TAMs into the antitumoral type, and those adding TAMs as targets in immunotherapies. Although exploring proper prognostic TAM markers or various combinations with other indicators of immune cells may seem to be a difficult task due to the diversity in TAMs, tumor subtypes, tumor node metastasis (TNM) stages, treating methods, and patient races, future studies will hopefully yield promising results.

At present, many undetermined problems existing in the study of TAMs in CRC remain to be elucidated. For instance, the effects of TAMs in human CRC progression are controversial, or even contradictory [55,165,167,168]. Numerous studies have shown that high density of macrophages is indicative of favorable outcome [164,165,169], while other data support the opposite finding [170]. Possible reasons for this include: (1) CD68 may also be expressed in stroma and even cancer cells on occasion, which implies that the data achieved from this marker should be examined carefully; (2) TAMs with complicated spatial locations (inside of or beside the CRC tissues) may have different functions; and (3) identification of phenotypic polarization and diversity is difficult [135].

With our improved understanding of TAM function and mechanisms in CRC, more promising therapies based on different principles are on the way. Recent studies have shown that TAMs can express PD-1, PD-L1, and myeloid-derived specific immune checkpoint signal regulatory protein-α (SIRPα) in the TME of CRC [171]. Therefore, immunotherapy targeting TAMs may synergistically enhance the efficacy of immunotherapy with immune checkpoint inhibitors such as anti-PD-1, PD-L1, and CTLA-4 antibodies, thereby enabling more CRC patients to benefit from immunotherapy. Additionally, those key candidates for reeducating TAMs, probiotics for maintaining the microbial homeostasis in the GI tract, and new TAM-targeted therapies based on the signaling pathways and functional positioning of TAM subtypes at different CRC stages can all be developed into prospective treatment strategies. Combined with conventional therapies such as chemotherapy, these new classes of remedies providing advances in efficacy and safety will surely benefit CRC patients in clinical practice.

## Figures and Tables

**Table 1:** Literature reports on the associations between TAMs and the prognosis of CRC patients.

Study Result	Expression in TAMs	Sample Size (Case)	Reference
Benign prognosis			
High-density CD68^+^ TAM subtypes in CRC tissues were significantly associated with good 5-year overall survival (OS) rates	High-density CD68^+^	6115	[155]
The ratio of CD68^+^ macrophages to colon cancer cells is associated with improved survival in colon cancer patients	High CD68^+^/colon cancer cells ratio	205	[156]
Adjuvant chemotherapy significantly improved recurrence-free survival (RFS) and OS for patients with high CD206^+^/CD68^+^ ratio of TAMs	High CD206^+^/CD68^+^ ratio	835	[157]
Both CD68^+^- and VEGF-expressing TAMs were predictive of improved survival rates in stage II and stage III colon cancer patients	CD68^+^ and VEGF	131	[158]
High infiltration of M1 macrophages is correlated with better prognosis in CRC in a stage-dependent manner	High M1^+^	485	[159]
Poor prognosis			
Infiltration of TAMs CD68^+^/iNOS^−^ in the tumor stroma is a negative prognostic factor	CD68^+^/iNOS^−^	89	[160]
An increased ratio of CD163^+^/CD68^+^ in the tumor invasive front (TF) was positively correlated with shorter CRC RFS and OS times	High CD163^+^/CD68^+^	81	[66]
An increase in the proportion of M2/M1 type TAMs was positively correlated with an increase in liver metastases in patients with colorectal cancer	High M2/M1 ratio	120	[161]
A decrease in the number of infiltrating CD68^+^ TAMs in the tumor stroma was associated with longer RFS and OS times in advanced CRC patients receiving bevacizumab combined with chemotherapy	High CD68^+^	123	[162]
The combination of FSP-1^+^ CAFs and CD163^+^ M2 TAMs was associated with poor survival rates more significantly than when these markers were studied alone	FSP-1^+^ CAFs and CD163^+^ M2 TAMs	289	[163]
